# Galectin-3/adiponectin as a new biological indicator for assessing the risk of type 2 diabetes: a cross-sectional study in a community population

**DOI:** 10.18632/aging.203101

**Published:** 2021-06-07

**Authors:** Diaozhu Lin, Xiaosi Hong, Kan Sun, Xiaoyun Zhang, Hong Lian, Jiahuan Wang, Na Mao, Xiuwei Zhang, Meng Ren, Li Yan, Feng Li, Lili You

**Affiliations:** 1 Department of Endocrinology, Sun Yat-Sen Memorial Hospital, Sun Yat-Sen University, Guangzhou 510120, People’s Republic of China; 2 Department of Endocrinology, Dongguan People's Hospital, Dongguan 523000, People’s Republic of China

**Keywords:** type 2 diabetes, age, galectin-3/adiponectin, diabetes risk assessment model

## Abstract

Objective: This study aimed to explore the association between the risk of newly diagnosed type 2 diabetes and galectin-3 and adiponectin and to investigate whether their joint action shows a favorable diabetes assessment performance.

Methods: We conducted a community-based study in 135 newly diagnosed patients with type 2 diabetes and 270 age- and sex-matched nondiabetic patients. Odds ratios and 95% confidence intervals were determined using logistic regression analysis. Receiver operating characteristic curve, decision curve analysis and calibration plot were used to explore their efficacy and clinical utility for models.

Results: High quartiles of galectin-3/adiponectin (quartile 4 vs 1: OR 2.43 [95% CIs: 1.21–5.00]) showed the strongest correlation with an increased risk of type 2 diabetes in the total population, which was consistent in the older population (age≥50 years old) in adjustment models. The combination + lipids + galectin-3/adiponectin model (AUC = 0.72 [95% CIs: 0.66-0.77]) displayed better diabetes assessment performance than the other two models.

Conclusions: High galectin-3 and low adiponectin levels were associated with the high risk of diabetes, and their joint action was a superior promising factor for evaluating diabetes risk. The diabetes discriminative strength of galectin-3/adiponectin was better in the older population than the younger.

## INTRODUCTION

Diabetes has been proven to be a chronic and complex metabolic disorder with severe health and socioeconomic burden worldwide [1]. Diabetes is characterized by chronic hyperglycemia and inflammatory diseases due to insulin resistance and dysfunction of insulin production [2]. According to the International Diabetes Federation (IDF), the number of patients with diabetes in the world is increasing, with an average global growth rate of 51% in 2019. It is estimated that the number of people with diabetes in the world will rise from 463 million to 700 million by 2045 [3]. The irreversibility of diabetes and its complications seriously reduce people’s quality of life and increase the death rate, and there is still no cure method available for diabetes. Consequently, it is of great significance to explore new assessment factors related to diabetes.

Galectin-3, a pleiotropic protein mainly secreted from macrophages involved in cell adhesion, differentiation, proliferation, migration, apoptosis, oxidative stress and the inflammatory response [4], plays an important role in promoting tissue fibrosis, such as renal damage and cardiac and vascular remodeling [5–7]. In addition, increased galectin-3 levels have been investigated in obese, prediabetic and diabetic populations [8–12]. To investigate how galectin-3 contributes to the development of diabetes, prior studies mostly focused on the correlation analysis between galectin-3 and various factors, especially glycosylated hemoglobin [8], insulin resistance [9, 10] and inflammatory factors [10, 12] but were deficient in further discussion of age. Meanwhile, Tsuyoshi et al. considered that galectin-3 is strongly correlated with adiponectin, which may jointly impact insulin sensitivity, indicating that the combination of galectin-3 and adiponectin may offer new opportunities for diabetes assessment [9].

Adiponectin, a 30-kDa hormone adipokine, is the only adipose-specific protein that is negatively regulated in obesity [13]. Previous studies have shown that adiponectin levels are inversely related to obesity, insulin resistance, metabolic syndrome, and progression from prediabetes to diabetes [14–16]. A meta-analysis also demonstrated that the inverse association between adiponectin and diabetes is accompanied by a dose-response relationship [17]. However, analysis using cubic splines revealed that the associations between adiponectin and new-onset diabetes were not linear, pointing to the correlation between adiponectin and type 2 diabetes being influenced by potential confounders such as age, sex and other metabolic factors [18]. Thus, the single utility and applicability of adiponectin for diabetes risk assessment may be limited, and prior investigations have proven that combination indices such as adiponectin/leptin [19] and homeostasis model assessment (HOMA)/adiponectin [20] play superior roles in assessing diabetes risk; in addition, the high-molecular weight (HMW)/total adiponectin ratio [18] showed a linear adjusted association with diabetes. However, previous investigations have not simultaneously analyzed the effect of changes in both galectin-3 and adiponectin concentrations on the risk of diabetes and insulin sensitivity and secretion.

Therefore, the purpose of our study was to further investigate the relationships among circulating galectin-3 and adiponectin on type 2 diabetes in a community population of different age groups and whether their joint action plays an outstanding role in diabetes risk assessment.

## RESULTS

According to galectin-3 quartiles, the characteristics of the participants were shown in Table 1. Those with higher plasma levels of galectin-3 were more likely to be older (P=0.001 for trend). Galectin-3 quartiles were also positively associated with body fat, systolic blood pressure (SBP) and fasting plasma glucose (FPG) (P<0.05).

**Table 1:** Clinical characteristics of participants according to quartiles of plasma level of galectin-3.

**Characteristic**	**Quartiles of plasma level of galectin-3 (range, ug/L)**				***P*** **for trend**	***P* values**
	**Q1** **[0.795, 3.74]** **n=101**	**Q2** **(3.74,4.80]** **n=101**	**Q3** **(4.80,5.88]** **n=101**	**Q4** **(5.88-14.10]** **n=101**		
Age(years)	50.95±11.31	51.82±11.01	55.68±11.67^#^	55.30±10.79^#^	**0.001**	**0.003**
Male(n,%)	58(57.43)	50(49.50)	49(48.51)	56(55.45)	0.755	0.506
Height(cm)	163.67±8.00	161.27±9.45	160.18±8.72^#^	160.45±9.63	**0.007**	**0.024**
Weight(kg)	66.55±11.91	65.52±13.62	64.98±11.81	64.37±12.58	0.203	0.647
BMI(kg/m^2^)	24.74±3.36	25.12±3.48	25.21±3.48	24.89±3.79	0.750	0.803
WC(cm)	85.72±10.49	85.77±10.11	87.00±9.99	86.65±10.27	0.373	0.755
HC(cm)	96.70±6.49	97.21±7.57	97.16±6.96	96.39±7.77	0.758	0.827
WHR	0.88±0.07	0.88±0.06	0.89±0.07	0.90±0.07	0.067	0.221
Bodyfat(%)	27.79±7.02	29.61±8.18	30.96±9.68^#^	28.81±6.79	0.218	**0.038**
SBP(mmHg)	120.4±11.22	123.9±15.53	127.3±19.50^#^	125.7±19.62	0.010	**0.026**
DBP(mmHg)	74.09±9.11	76.24±9.96	76.54±11.69	76.54±11.1^7#^	0.102	0.281
TC(mmol/L)	5.41±1.00	5.41±1.15	5.29±1.08	5.56±1.18	0.489	0.385
TG(mmol/L)	1.40(1.00-1.99)	1.43(0.99-2.29)	1.52(1.10-2.22)	1.52(1.13-2.26)^#^	0.075	0.364
HDL-C (mmol/L)	1.39(1.19-1.70)	1.38(1.15-1.61)	1.40(1.23-1.59)	1.36(1.16-1.56) ^#^	0.060	0.240
LDL-C(mmol/L)	3.21±0.97	3.26±1.96	3.11±1.02	3.29±0.89	0.825	0.576
FPG(mmol/L)	5.26(4.70-5.90)	5.60(5.03-6.35)	5.20(4.77-5.96)	5.38(4.82-6.80^)^	0.224	**0.022**
HbA1c (%)	5.72(5.42-6.11)	5.81(5.52-6.51)	5.62(5.32-6.31)	5.81(5.42-6.60)	0.587	0.264
Adiponectin(mg/L)	4.10(2.60-5.90)	3.70(2.30-5.50)	4.10(2.80-6.10)	3.60(2.60-5.20)	0.446	0.227

*p<0.001 compared with galectin-3 Q1 ([0.795, 3.74]) group. # p<0.05 compared with galectin-3 Q1 ([0.795, 3.74]) group.

$ p values were for the ANOVA or χ^2^ analyses across the three groups.

**Figure 1:**
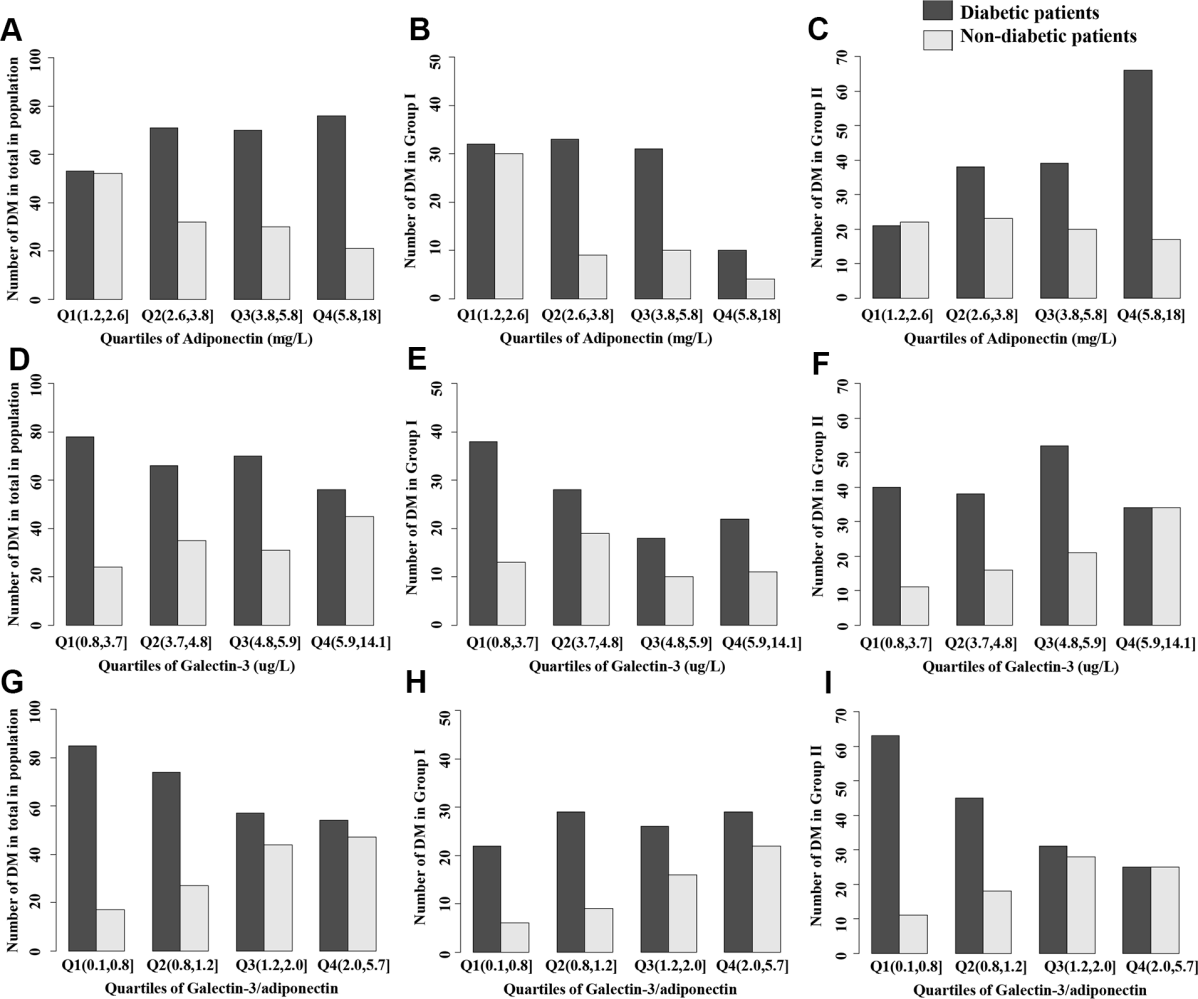
**Distribution of diabetes in total population, Group I or Group II stratified by quartiles of biomarkers.** (**A**–**C**) Adiponectin, (**D**–**F**) Galectin-3; (**G**–**I**) Galectin-3/adiponectin. Group I: population with the age< 50 years old; Group II: population with the age≥50 years old.

Demographic and biochemical findings of subjects with and without incident type 2 diabetes in the total population, Group I (age<50 years old) and Group II (age≥50 years old) were expressed in Table 2. In the total population, compared to the nondiabetic group, body weight, body mass index (BMI), waist circumference (WC), waist-hip ratio (WHR), body fat, diastolic blood pressure (DBP), total cholesterol (TC), triglycerides (TG), galectin-3 and galectin-3/adiponectin (Gal-3/ADPN) of the diabetic group were higher (P<0.05), and high-density lipoprotein cholesterol (HDL-C) and adiponectin were lower (P<0.05). We found that the differences in these factors were mainly concentrated in the older group rather than the younger group. Higher galectin-3 and galectin-3/adiponectin quartiles were more significantly associated with a greater number of diabetic patients and fewer nondiabetic patients in group I than in group II (Figure 1).

**Table 2:** Characteristics of subjects with and without incident type 2 diabetes.

**Variables**	**Total population (405)**			**Group I (Age<50y, n=159)**			**Group II (Age≥50y, n=246)**		
	**Non-DM (270)**	**T2DM (135)**	**P values**	**Non-DM (106)**	**T2DM (53)**	**P values**	**Non-DM (164)**	**DM (82)**	**P values**
Age(years)	53.42±11.35	53.42±11.37	0.998	41.89±6.01	41.89±6.04	1.00	60.87±6.84	60.88±6.88	0.995
Male(n)	142	71	1.000	33	20	1.00	38	44	1.000
Height(cm)	161.44±8.62	161.31±9.84	0.898	165.14±8.38	164.75±8.11	0.779	159.05±7.92	159.09±10.26	0.975
Weight(kg)	64.25±12.11	67.60±12.92	**0.013**	68.98±13.46	72.21±14.48	0.178	61.20±10.06	64.62±10.89	**0.018**
BMI(kg/m2)	24.54±3.56	25.90±3.89	**<0.001**	25.20±3.95	26.45±4.06	0.068	24.12±3.22	25.54±3.76	**0.004**
WC(cm)	85.20±10.06	88.44±10.11	**0.003**	85.45±11.15	88.58±10.91	0.093	85.03±9.32	88.34±9.62	**0.011**
HC(cm)	96.42±7.03	97.78±7.45	0.080	97.42±7.38	98.55±7.54	0.369	95.78±6.74	97.28±7.39	0.124
WHR	0.88±0.07	0.90±0.07	**0.003**	0.87±0.07	0.90±0.07	0.054	0.89±0.07	0.91±0.06	**0.021**
Bodyfat(%)	28.45±7.18	31.02±9.36	**0.005**	26.99±7.10	30.16±11.52	0.070	29.39±7.09	31.58±7.67	**0.032**
SBP(mmHg)	123.14±15.77	126.63±18.92	0.066	118.83±12.84	120.42±15.10	0.517	125.91±16.86	130.65±20.11	0.069
DBP(mmHg)	74.60±9.88	78.35±11.36	**0.001**	74.38±10.42	76.38±10.81	0.268	74.75±9.54	79.62±11.59	**0.001**
TC(mmol/L)	5.30±1.07	5.64±1.14	**0.005**	5.08±1.09	5.60±1.15	**0.007**	5.44±1.04	5.66±1.14	0.152
TG(mmol/L)	1.38(0.93-1.92)	1.78(1.27-2.62)	**<0.001**	1.35(0.94-2.06)	1.90(1.38-2.76)	**<0.001**	1.40(0.93-1.85)	1.61(1.20-2.60)	**<0.001**
HDL-C (mmol/L)	1.41(1.20-1.65)	1.33(1.12-1.57)	**0.013**	1.32(1.17-1.59)	1.28(1.02-1.50)	0.115	1.45(1.21-1.69)	1.35(1.17-1.61)	**0.046**
LDL-C(mmol/L)	3.16±0.94	3.33±1.01	0.102	3.01±0.90	3.24±1.04	0.174	3.25±0.95	3.38±0.99	0.319
FPG(mmol/L)	5.06(4.66-5.52)	7.00(5.90-8.49)	**<0.001**	5.10(4.70-5.56)	7.50(6.20-9.20)	**<0.001**	5.02(4.65-5.50)	6.82(5.70-7.73)	**<0.001**
HbA1c (%)	5.52(5.32-5.81)	6.70(6.51-7.40)	**<0.001**	5.47(5.22-5.72)	6.70(6.51-8.29)	**<0.001**	5.62(5.37-5.91)	6.70(6.51-7.30)	**<0.001**
Adiponectin(mg/L)	4.10(3.00-6.20)	3.20(2.10-4.60)	**<0.001**	3.50(2.40-4.30)	2.40(1.80-4.00)	**0.003**	5.05(3.40-7.00)	3.70(2.50-5.30)	**0.0002**
Galectin-3(ug/L)	4.78±1.77	5.34±1.76	**0.003**	4.65±1.83	4.85±1.78	0.514	4.86±1.73	5.66±1.69	**0.0006**
Gal-3/ADPN(10-3)	1.03(0.70-1.74)	1.65(1.12-2.34)	**<0.001**	1.25(0.87-2.04)	1.83(1.17-2.46)	**0.006**	0.94(0.60-1.53)	1.41(1.08-2.13)	**<0.001**

Non-DM: non-diabetes mellitus; T2DM: type 2 diabetes mellitus; Group I: population with the age< 50 years old; Group II: population with the age≥ 50 years old.

Gal-3/ADPN:galectin-3/adiponectin.

We further studied the prevalence of diabetes and biomarkers of different quartiles in three groups. After adjustments for potential confounding factors, the approximate gradient associations between diabetes risk and adiponectin (negative), galectin-3 (positive) and galectin-3/adiponectin (positive) were consistently detected in both univariable and multivariable logistic regression analyses (Tables 3, 4). After accounting for BMI, SBP and TG, compared with adiponectin (e.g., quartile 4 vs 1: odds ratio (OR): 0.51, [95% confidence interval (95% CI): 0.26–1.02]; P=0.115) and galectin-3 (e.g., quartile 4 vs 1: OR 2.37 [95% CI: 1.27–4.53]; P=0.021) alone, participants of the total population in high quartiles of galectin-3/adiponectin (e.g., quartile 4 vs 1: OR 2.43 [95% CI: 1.21–5.00]; P=0.005) also showed a strong correlation with increased odds of diabetes, which can be validated in group II (e.g., quartile 4 vs 1: OR 3.28 [95% CI: 1.32–8.41]; P=0.003) but not in group I (e.g., quartile 4 vs 1: OR 1.33 [95% CI: 0.42–4.46]; P=0.50505) (Table 4).

**Table 3:** Association of increased adiponectin, galectin-3 and galectin-3/adiponectin with risk of prevalent diabetes.

**Variables**	**1-Quartile change of biomarker parameters**			**AUC(95%CI)**	**P values**
	**Model 1**	**Model 2**	**Model 3**		
Total population					
Adiponectin	0.67(0.55-0.82)	0.71(0.58-0.87)	0.84(0.67-1.04)	0.684(0.628-0.740)	-
Galectin-3	1.31(1.09-1.58)	1.30(1.07-1.58)	1.27(1.04-1.55)	0.695(0.641-0.749)	0.476
Galectin-3/adiponectin	1.65(1.36-2.02)	1.57(1.28-1.92)	1.36(1.10-1.70)	0.700(0.645-0.755)	0.101
Group I(Age<50y)					
Adiponectin	0.65(0.45-0.92)	0.69(0.47-0.99)	0.88(0.58-1.34)	0.698(0.609-0.787)	-
Galectin-3	1.11(0.83-1.49)	1.11(0.82-1.50)	1.09(0.79-1.49)	0.701(0.612-0.789)	0.778
Galectin-3/adiponectin	1.46(1.07-2.03)	1.41(1.02-1.97)	1.13(0.79-1.63)	0.697(0.608-0.787)	0.913
Group II(Age≥50y)					
Adiponectin	0.65(0.50-0.83)	0.70(0.54-0.90)	0.79(0.60-1.04)	0.681(0.609-0.753)	-
Galectin-3	1.50(1.16-1.95)	1.46(1.13-1.92)	1.42(1.08-1.87)	0.700(0.632-0.767)	0.460
Galectin-3/adiponectin	1.82(1.41-2.37)	1.69(1.30-2.23)	1.53(1.16-2.04)	0.712(0.643-0.780)	**0.056**

Model 1:Unadjusted model.

Model 2:Adjusted by BMI and SBP.

Model 3:Adjusted by BMI, SBP and TG.

The values of AUC and 95% CI were analyzed according to the results of Model 3, and *p* value is the comparison of the corresponding ROC curve area.

**Table 4:** Adjusted odds ratios for diabetes by quartiles of adiponectin`galectin-3 and galectin-3/adiponectin.

	**Quartile 1**	**Quartile 2**	**Quartile 3**	**Quartile 4**	**P values**
Total population					
Adiponectin	1.00	0.60(0.33-1.09)	0.80(0.42-1.52)	0.51(0.26-1.02)	0.115
Galectin-3	1.00	1.60(0.84-3.06)	1.22(0.63-2.38)	2.37(1.27-4.53)	**0.021**
Galectin-3/adiponectin	1.00	1.55(0.77-3.17)	2.77(1.42-5.57)	2.43(1.21-5.00)	**0.005**
Group I (Age<50y)					
Adiponectin	1.00	0.47(0.17-1.20)	0.70(0.26-1.84)	0.88(0.18-3.79)	0.563
Galectin-3	1.00	1.93(0.78-4.89)	1.24(0.41-3.67)	1.45(0.51-4.08)	0.615
Galectin-3/adiponectin	1.00	0.92(0.27-3.30)	1.33(0.42-4.53)	1.33(0.42-4.46)	0.508
Group II (Age≥50y)					
Adiponectin	1.00	0.70(0.30-1.63)	0.86(0.35-2.11)	0.45(0.18-1.08)	0.096
Galectin-3	1.00	1.35(0.54-3.46)	1.21(0.50-3.98)	3.00(1.30-7.31)	**0.012**
Galectin-3/adiponectin	1.00	2.03(0.87-4.90)	4.00(1.75-9.60)	3.28(1.32-8.41)	**0.003**

All of models were adjusted by BMI, SBP and TG.

**Figure 2:**
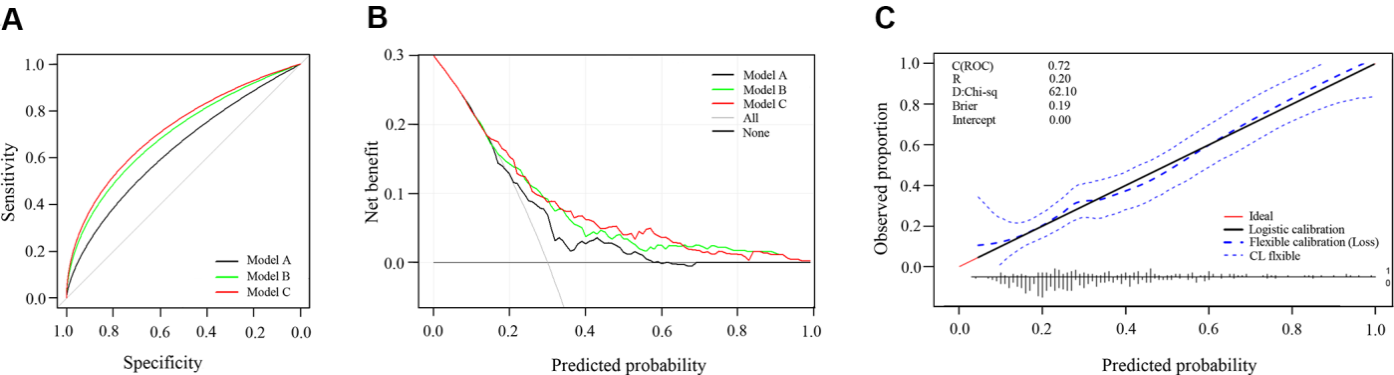
**Predictive accuracy of three models to assess the risk of type 2 diabetes.** (**A**) Receiver operating characteristic curve of three models; (**B**) Decision curve analysis of three models; (**C**) Calibration curve of combination+lipids+galectin-3/adiponectin model. Model A: combination model (including the noninvasive factors BMI, WC, body fat and DBP) Model B: combination + lipids model (additionally including the invasive screening factors TC, TG and HDL-C) Model C: combination + lipids + galectin-3/adiponectin model (finally including galectin-3/adiponectin).

We constructed the following three diabetes risk assessment models: combination model (Model A) (including the noninvasive factors BMI, WC, body fat and DBP), combination + lipids model (Model B) (additionally including the invasive screening factors TC, TG and HDL-C) and combination + lipids + galectin-3/adiponectin model (Model C) (finally including galectin-3/adiponectin). These models’ discrimination could be confirmed by receiver operating characteristic (ROC) curve analysis (Figure 2A). ROC curve analysis showed that galectin-3/adiponectin had the best discrimination accuracy for diabetes, especially in Model 3 of group II when compared with adiponectin (area under curve (AUC)=0.712 [95% CI: 0.643–0.780]; P=0.056). The combination + lipids + galectin-3/adiponectin model (AUC = 0.72 [95% CI: 0.66-0.77]) displayed better assessed performance than the combination model and the combination + lipids model (AUC =0.64 [95% CI: 0.58–0.69] and AUC = 0.70 [95% CI: 0.65– 0.76], respectively). Meanwhile, net reclassification improvement (NRI) and integrated discrimination improvement (IDI) can also depict significantly improved assessed performance for the combination + lipids + galectin-3/adiponectin model (NRI=0.5481 [95% CI: 0.3486–0.7477], P<0.001; IDI=0.0957 [95% CI: 0.0616-0.1299], P<0.001) compared with the combination model, suggesting distinct model improvement. To explore their clinical utility for prediction errors in risk assessment models, we built a decision analysis curve for the assessment of three models. Compared with other models, the combination + lipids + galectin-3/adiponectin model had a higher net benefit over the whole range of threshold probability, the difference being sizeable and visible on the graph for values of probability between 0.15 and 0.65 (Figure 2B). Finally, the prediction results and diagonals of the combination + lipids + galectin-3/adiponectin model were basically coincident, which showed that the calibration curve had good concordance between the predicted probability and actual probability, with a mean absolute error of 0.024 (Figure 2C).

## DISCUSSION

In this study, we demonstrated that high galectin-3 and low adiponectin levels were clinically associated with type 2 diabetes, and their joint action played an outstanding role in diabetes risk assessment. The combination + lipids + galectin-3/adiponectin model displayed favorable performance in diabetes risk assessment. Meanwhile, this investigation yielded novel findings with regard to the positive galectin-3/adiponectin diabetes association in an older population.

Analyses of adiponectin concentrations exhibited, as expected, an inverse correlation with diabetes, which was consistent with previous studies [17]. High adiponectin levels in the blood are negatively correlated with body weight, abdominal obesity, and insulin resistance, which can significantly improve the ability to predict the risk of diabetes [21]. Different from the dynamic criteria for diabetes of blood glucose and glycosylated hemoglobin, the lower adiponectin levels in healthy individuals, obese people or prediabetes can serve as predictors to screen out the high-risk population with normal blood glucose but abnormal physiological metabolism and dynamically reflect the level of insulin resistance and individual metabolic health [21]. Thus, adiponectin plays a significant role in screening out populations with high diabetes risk so that intervention can occur early to reverse the progression of type 2 diabetes.

In addition, in contrast to most published studies merely focusing on the elderly population [18, 22, 23], Yoshinari et al. found a significant positive association between serum adiponectin levels and age in healthy subjects and patients with diabetes in both men and women, which was independent of glucose metabolism and lipid profiles [24]. Thus, we further conducted age stratified analyses to determine that the inverse association between adiponectin and type 2 diabetes tended to be stronger in older adults than in younger adults. A presumption for the age-related increase in serum adiponectin levels is that the population with high-level adiponectin lives long, and consequently, the elderly population could have high concentrations of adiponectin [24]. Another plausible interpretation is positive feedback due to the downregulation or resistance of adiponectin receptors with age, but we could not measure adiponectin receptors in this study to investigate this possibility. As a result, the relationship between serum adiponectin and age is not well defined, and basic research is required to explain the clinical findings. On the basis of these results, it is of great significance to take into account age-related changes in serum adiponectin levels when interpreting people’s serum adiponectin levels in clinical practice.

Simultaneously, prior clinical studies had proven our results that higher galectin-3 concentrations are associated with the development of obesity and type 2 diabetes [8–12]. In epidemiological studies, patients with prediabetes or type 2 diabetes exhibited elevated systemic galectin-3, which correlates inversely with glycosylated hemoglobin [8] and positively with insulin sensitivity [9], supporting that galectin-3 is more likely to be an alternative biomarker in the progression of diabetes. In a large, multiethnic study conducted in Dallas, galectin-3 was associated with diabetes prevalence and incidence, possibly through the inflammatory pathway contributing to B-cell fibrosis and impaired insulin secretion [12]. Additionally, growing evidence indicated that galectin-3 had a positive correlation with age [11, 12], which was also validated in our work (P=0.001 for trend). Nevertheless, concrete discussion regarding galectin-3 and age remains undetermined. It is hypothesized that the elderly population always possesses decreased body immunity, aging organ function, a longer course of type 2 diabetes and more complicated diseases. Thus, the worse adaptive regulatory capacity is accompanied by the more obviously different pathophysiologic performance when diabetes occurs. Therefore, further investigation of the underlying mechanisms, focusing on their isomer distribution, is needed to elucidate the associations with age. Our study further explored the relationship between galectin-3 and glucose metabolism in different age groups, which was more obvious in the elderly population. It had been suggested that galectin-3 may be involved in the important process of abnormal glucose metabolism in the old population, serving as an independent evaluation index of glucose metabolism in the elderly. These findings strongly support the hypothesis that galectin-3 and adiponectin may play critical roles in the pathogenesis of aging and abnormal glucose metabolism. Therefore, to optimize the accuracy of assessing diabetes risk, age differences may need to be taken into account with respect to galectin-3 and adiponectin in clinical application.

In the clinical, the albumin/globulin (A/G), aspartate aminotransferase/alanine aminotransaminase(AST/ALT) and free prostate specific antigen/ total prostate specific antigen (FPSA/TPSA) ratios have been proven to have a “1+1>2” effect compared with the single index. In addition to leptin/adiponectin [19], which had been widely proposed to be a sensitive indicator for evaluating diabetes risk, the ratios of HMW/total adiponectin [18], HOMA/adiponectin [20] and adiponectin/ferritin [25] were also applied for assessing diabetes. We innovatively explored whether the combination of two biomarkers can play a preferable role in diabetes risk assessment. As the multiple regression analysis shows, a high level of galectin-3/adiponectin showed a strong correlation with increased odds of diabetes, and the ROC curve showed that galectin-3/adiponectin had favorable discrimination accuracy for diabetes, especially in the elderly after adjusting for confounding factors. To screen and intervene in diabetes in a timely and effective manner, the establishment of a risk assessment model is of great significance.

Additionally, there are several reasons why the level of galectin-3/adiponectin may be superior to a single parameter in the assessment of increasing diabetes incidence. Although two biomarkers show distinct tendencies in obesity and diabetes, growing evidence has revealed that galectin-3 and adiponectin seem to have particular relevance in the pathogenesis of diabetes. First, both of them were elevated in the older population with obesity and diabetes, indicating their critical clinical application in the elderly. Second, since diabetes is a chronic inflammatory disease, galectin-3 and adiponectin may act as inflammatory factors that are directly involved in insulin resistance and diabetic complications. Increasing evidence suggests that the two biomarkers may protectively mediate internal glycometabolism by triggering insulin and inflammation pathways. Moreover, it is hypothesized that galectin-3, mainly derived from macrophages and the adipokine adiponectin, might promisingly participate in the “cross-talk” of macrophages and adipocytes, serving as a new diabetic target by functioning together with insulin resistance and inflammation [26, 27]. This study did shed more light on the potential role of galectin-3/adiponectin in the pathophysiology of diabetes; thus, future studies are warranted to further characterize the link between galectin-3/adiponectin and diabetes.

Several limitations require consideration in our study. Firstly, whether our results can be generalized to other ethnic groups was not ascertained because the present study was conducted exclusively in Guangdong community residents in China, yet the previous Multi-Ethnic Study has demonstrated that there are consistent associations of galectin-3 and adiponectin with diabetes across ethnic groups. Secondly, this cross-sectional study failed to determine the causality or temporal relationship among galectin-3, adiponectin and diabetes development. Besides, several confounding factors were not taken into account here, such as exercise, diet, and lifestyle, and incomplete data compilation may influence the interpretation of the result of this study. Thus, large-scale prospective cohort studies should be considered to conduct to strength the findings of the present study. To some extent, the population in the present study was still a convenience sample, and selection bias is inevitable. The relatively small size of our sample population also fails to meet model validation criteria; thus, external studies are necessary to verify our findings in large-scale prospective cohort studies. Fourth, FPG and HAb1c were used for the diagnosis of diabetes in our study, and the lack of OGTT as the diabetes diagnostic criteria will reduce the prevalence of diabetes. However, FPG and HAb1c as the diagnostic criteria of diabetes were commonly used in large-scale epidemiological investigation [18, 22, 28]. Thus, it’s suggested that our study still show a favorable application prospect, and provide the basis for the follow-up scientific research. Fifth, it’s difficult to distinguish those who with higher galectin-3 results from the gene expressions diversification without displaying any diagnosed disorders in the early stage. Thus, related follow-up study and gene detection in our subsequent study design is needed. In addition, our assay of total adiponectin failed to investigate different forms of adiponectin, such as high-molecular weight adiponectin, which may play distinct roles in the regulation of diabetes.

In conclusion, high galectin-3 and low adiponectin levels were associated with the risk of type 2 diabetes, and our findings further add evidence that their joint action may be a superior promising parameter for evaluating diabetes risk. Meanwhile, the discriminative strength of the galectin-3/adiponectin ratio for diabetes was better in the older population. At present, the Finland FINDRISC noninvasive model [29] and American Framingham invasive model [30] are widely used. The combination + lipids + galectin-3/adiponectin model was proved to have good discrimination and clinical efficacy, which provided a new entry point for the establishment of a diabetes risk assessment model in the future.

## MATERIALS AND METHODS

### Study populations

We performed a cross-sectional study in Guangzhou and Dongguan, China, from December 2018 to October 2019. Participants were recruited from the Sun Yat-sen Memorial Hospital of Physical Examination Center in Guangzhou and Dongguan communities (Dalingshan community, Zhangmu community, Daojiao community, Qiaotou community, Songshan lake community, Qingxi community, Zhang'an community and Meinian Physical Examination Center). During the recruiting phase, a total of 3866 residents who were Han ethnicity of the Chinese population, aged 18-70 years, and lived in those regions ≥3 years were invited to participate. Subjects who met the following criteria were excluded sequentially from the analyses: (1) pregnant women; (2) those who suffered from mental illness or severe physical diseases such as hepatic cirrhosis, chronic renal failure or evident cardiac insufficiency; (3) those with a history of infectious disease or malignant tumors; (4) those who were diagnosed with hypertension, hyperlipidemia, cardiovascular and cerebrovascular diseases; (5) those with other endocrine diseases; and (6) those who used drug or dietary supplements or functional food long term (≥3 times/week for more than 3 months). We further excluded participants who failed to provide blood glucose information (n=171) and with a history or family history of diabetes (n=13). In addition, individuals who failed to provide information (age, n=10; FPG, n=2; BMI, n=43; SBP, n=105; TG, n=14; WC, n=195 or body fat, n=218) were excluded.

**Figure 3:**
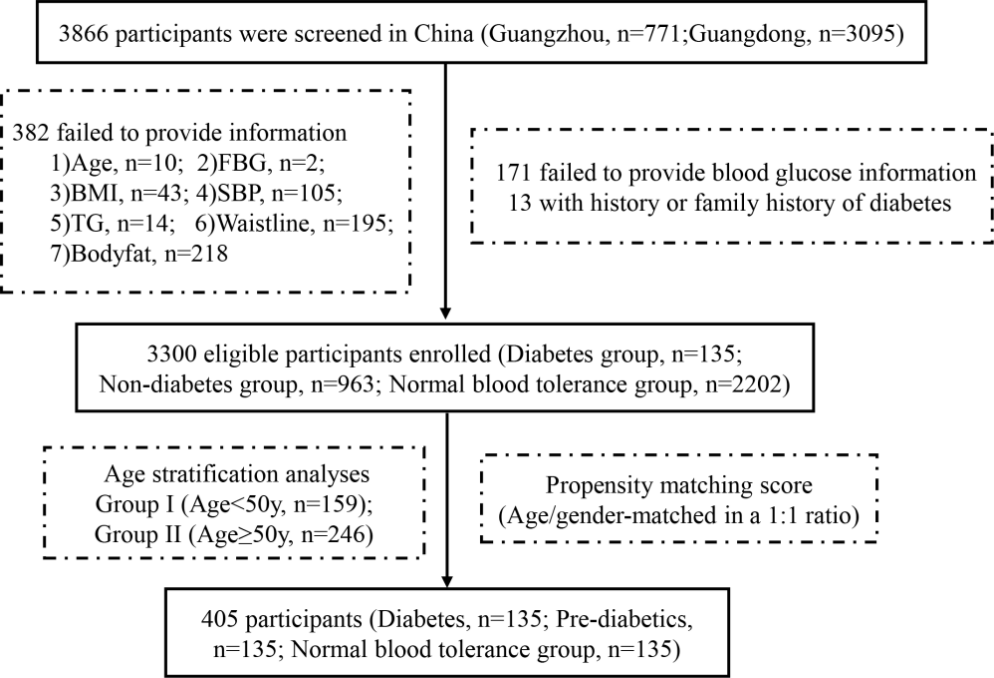
Flow diagram of participants in the study.

A total of 3300 individuals were included in the final data analyses, including 135 individuals with newly diagnosed type 2 diabetes (FPG≥7.0 mmol/L or glycosylated hemoglobin (HbA1c) ≥6.5%) [31]. By matching participants’ sex and age, 135 prediabetics and 135 healthy controls were selected from the prediabetes group and normal glucose tolerance group, respectively. Finally, 405 individuals underwent galectin-3 and adiponectin measurements, and the participants were also divided into group I (age<50y, n=159) and group II (age≥50y, n=246) for further analysis. The details can be seen in the flow diagram (Figure 3). This study was approved by the Ethics Committee of the Faculty of Sun Yat-sen Memorial Hospital affiliated with Sun Yat-sen University. Written informed consent was obtained from all participants.

### Data collection and measurements

All participants completed anthropometrical measurements with the assistance of trained staff using standard protocols. They were required to remove their shoes and wear light clothing during the measurements of body height and weight. BMI was calculated as weight in kilograms divided by height in meters squared (kg/m^2^). Measurements of WC and hip circumference (HC) required the subjects to stand and breathe steadily. WHR is calculated as the ratio of WC to HC. After standing evenly on both feet and inputting participants’ information (including sex, age, height and weight) according to the operation instructions, body fat content was measured by the family fat scale (Novo Nordisk, Denmark). After at least 5 min of seated rest, both systolic and diastolic blood pressure were measured twice with automatic electronic equipment (OMRON, Omron Company, Japan), and the average value was used for subsequent analyses.

After overnight fasting for at least 10 hours, venous blood samples were collected, centrifuged, and immediately stored at -80° C for laboratory tests until the assays were performed. An autoanalyzer (Beckman CX-7 Biochemical Autoanalyser, Brea, CA, USA) was used to measure FPG, TC, TG, low-density lipoprotein cholesterol (LDL-C) and HDL-C. High-performance liquid chromatography (Bio-Rad, Hercules, CA) was used to assess HbA1c. Enzyme-linked immunosorbent assay (Automated ELISA System, DYNEX, USA) was used to measure the serum level of galectin-3. A latex enhanced turbidimetric immunoassay (BS-600 automatic biochemical instrument, Mindray, China) was used to measure the serum level of adiponectin.

### Statistical analysis

Continuous variables were expressed as the mean ± standard deviation (data with normal distribution) or medians (data with nonnormal distribution). TG, HDL-C, FPG, HbA1c, adiponectin and galectin-3 with a nonnormal distribution were logarithmically transformed before analysis. Differences between groups were tested by one-way ANOVA, and post hoc comparisons were performed by using Bonferroni correction. Categorical variables were expressed as numbers (proportions), and differences among groups were analyzed with chi-square tests. Logistic regression was used to calculate odds ratios and 95% confidence intervals. The unadjusted and multivariate-adjusted logistic regression models were used to assess the influencing factors that increased the risk of diabetes. Model 1 was unadjusted. Model 2 was adjusted by BMI and SBP. Model 3 was further adjusted by TG. We constructed three diabetes risk assessment models. The discriminative ability of different models was examined through the area under the receiver operating characteristic curve. Decision curve analysis was carried out to compare the potential net benefit of the assessment models. A calibration plot was used to assess the calibration of the model. Propensity score matching was performed by matching the function of the MatchIt package in RStudio, matching subgroups with similar sex and age to the group of newly diagnosed patients with diabetes. RStudio version 3.6.1 was used for all statistical analyses. Statistical tests were two-sided, and differences were considered to be statistically significant when p < 0.05.

## References

[1] Zheng Y, Ley SH, Hu FB. Global aetiology and epidemiology of type 2 diabetes mellitus and its complications. Nat Rev Endocrinol. 2018; 14:88–98. 10.1038/nrendo.2017.151 29219149

[2] Donath MY, Shoelson SE. Type 2 diabetes as an inflammatory disease. Nat Rev Immunol. 2011; 11:98–107. 10.1038/nri2925 21233852

[3] Saeedi P, Petersohn I, Salpea P, Malanda B, Karuranga S, Unwin N, Colagiuri S, Guariguata L, Motala AA, Ogurtsova K, Shaw JE, Bright D, Williams R, and IDF Diabetes Atlas Committee. Global and regional diabetes prevalence estimates for 2019 and projections for 2030 and 2045: Results from the International Diabetes Federation Diabetes Atlas, 9^th^ edition. Diabetes Res Clin Pract. 2019; 157:107843. 10.1016/j.diabres.2019.107843 31518657

[4] Pugliese G, Iacobini C, Ricci C, Blasetti Fantauzzi C, Menini S. Galectin-3 in diabetic patients. Clin Chem Lab Med. 2014; 52:1413–23. 10.1515/cclm-2014-0187 24940712

[5] Tan KC, Cheung CL, Lee AC, Lam JK, Wong Y, Shiu SW. Galectin-3 is independently associated with progression of nephropathy in type 2 diabetes mellitus. Diabetologia. 2018; 61:1212–19. 10.1007/s00125-018-4552-z 29417184

[6] Harrison SA, Marri SR, Chalasani N, Kohli R, Aronstein W, Thompson GA, Irish W, Miles MV, Xanthakos SA, Lawitz E, Noureddin M, Schiano TD, Siddiqui M, et al. Randomised clinical study: GR-MD-02, a galectin-3 inhibitor, vs. placebo in patients having non-alcoholic steatohepatitis with advanced fibrosis. Aliment Pharmacol Ther. 2016; 44:1183–98. 10.1111/apt.13816 27778367

[7] Ho JE, Liu C, Lyass A, Courchesne P, Pencina MJ, Vasan RS, Larson MG, Levy D. Galectin-3, a marker of cardiac fibrosis, predicts incident heart failure in the community. J Am Coll Cardiol. 2012; 60:1249–56. 10.1016/j.jacc.2012.04.053 22939561 PMC3512095

[8] Weigert J, Neumeier M, Wanninger J, Bauer S, Farkas S, Scherer MN, Schnitzbauer A, Schäffler A, Aslanidis C, Schölmerich J, Buechler C. Serum galectin-3 is elevated in obesity and negatively correlates with glycosylated hemoglobin in type 2 diabetes. J Clin Endocrinol Metab. 2010; 95:1404–11. 10.1210/jc.2009-1619 20080851

[9] Ohkura T, Fujioka Y, Nakanishi R, Shiochi H, Sumi K, Yamamoto N, Matsuzawa K, Izawa S, Ohkura H, Ueta E, Kato M, Miyoshi E, Taniguchi S, Yamamoto K. Low serum galectin-3 concentrations are associated with insulin resistance in patients with type 2 diabetes mellitus. Diabetol Metab Syndr. 2014; 6:106. 10.1186/1758-5996-6-106 25302080 PMC4190474

[10] Yilmaz H, Cakmak M, Inan O, Darcin T, Akcay A. Increased levels of galectin-3 were associated with prediabetes and diabetes: new risk factor? J Endocrinol Invest. 2015; 38:527–33. 10.1007/s40618-014-0222-2 25501605

[11] Atalar MN, Abuşoğlu S, Ünlü A, Tok O, İpekçi SH, Baldane S, Kebapcılar L. Assessment of serum galectin-3, methylated arginine and Hs-CRP levels in type 2 diabetes and prediabetes. Life Sci. 2019; 231:116577. 10.1016/j.lfs.2019.116577 31211997

[12] Vora A, de Lemos JA, Ayers C, Grodin JL, Lingvay I. Association of Galectin-3 With Diabetes Mellitus in the Dallas Heart Study. J Clin Endocrinol Metab. 2019; 104:4449–58. 10.1210/jc.2019-00398 31162551

[13] Kadowaki T, Yamauchi T, Kubota N, Hara K, Ueki K, Tobe K. Adiponectin and adiponectin receptors in insulin resistance, diabetes, and the metabolic syndrome. J Clin Invest. 2006; 116:1784–92. 10.1172/JCI29126 16823476 PMC1483172

[14] Abdella NA, Mojiminiyi OA. Clinical Applications of Adiponectin Measurements in Type 2 Diabetes Mellitus: Screening, Diagnosis, and Marker of Diabetes Control. Dis Markers. 2018; 2018:5187940. 10.1155/2018/5187940 30069271 PMC6057311

[15] Kim JY, Ahn SV, Yoon JH, Koh SB, Yoon J, Yoo BS, Lee SH, Park JK, Choe KH, Guallar E. Prospective study of serum adiponectin and incident metabolic syndrome: the ARIRANG study. Diabetes Care. 2013; 36:1547–53. 10.2337/dc12-0223 23275369 PMC3661834

[16] Tabák AG, Carstensen M, Witte DR, Brunner EJ, Shipley MJ, Jokela M, Roden M, Kivimäki M, Herder C. Adiponectin trajectories before type 2 diabetes diagnosis: Whitehall II study. Diabetes Care. 2012; 35:2540–47. 10.2337/dc11-2263 22933430 PMC3507593

[17] Li S, Shin HJ, Ding EL, van Dam RM. Adiponectin levels and risk of type 2 diabetes: a systematic review and meta-analysis. JAMA. 2009; 302:179–88. 10.1001/jama.2009.976 19584347

[18] Kizer JR, Arnold AM, Benkeser D, Ix JH, Djousse L, Zieman SJ, Barzilay JI, Tracy RP, Mantzoros CS, Siscovick DS, Mukamal KJ. Total and high-molecular-weight adiponectin and risk of incident diabetes in older people. Diabetes Care. 2012; 35:415–23. 10.2337/dc11-1519 22148099 PMC3263897

[19] Jung CH, Rhee EJ, Choi JH, Bae JC, Yoo SH, Kim WJ, Park CY, Mok JO, Kim CH, Lee WY, Oh KW, Park SW, Kim SW. The relationship of adiponectin/leptin ratio with homeostasis model assessment insulin resistance index and metabolic syndrome in apparently healthy Korean male adults. Korean Diabetes J. 2010; 34:237–43. 10.4093/kdj.2010.34.4.237 20835341 PMC2932893

[20] Vilela BS, Vasques AC, Cassani RS, Forti AC, Pareja JC, Tambascia MA, Geloneze B, and BRAMS Investigators. The HOMA-Adiponectin (HOMA-AD) Closely Mirrors the HOMA-IR Index in the Screening of Insulin Resistance in the Brazilian Metabolic Syndrome Study (BRAMS). PLoS One. 2016; 11:e0158751. 10.1371/journal.pone.0158751 27490249 PMC4973901

[21] Spranger J, Kroke A, Möhlig M, Bergmann MM, Ristow M, Boeing H, Pfeiffer AF. Adiponectin and protection against type 2 diabetes mellitus. Lancet. 2003; 361:226–28. 10.1016/S0140-6736(03)12255-6 12547549

[22] Wannamethee SG, Lowe GD, Rumley A, Cherry L, Whincup PH, Sattar N. Adipokines and risk of type 2 diabetes in older men. Diabetes Care. 2007; 30:1200–05. 10.2337/dc06-2416 17322479

[23] Snijder MB, Heine RJ, Seidell JC, Bouter LM, Stehouwer CD, Nijpels G, Funahashi T, Matsuzawa Y, Shimomura I, Dekker JM. Associations of adiponectin levels with incident impaired glucose metabolism and type 2 diabetes in older men and women: the hoorn study. Diabetes Care. 2006; 29:2498–503. 10.2337/dc06-0952 17065691

[24] Tomono Y, Hiraishi C, Yoshida H. Age and sex differences in serum adiponectin and its association with lipoprotein fractions. Ann Clin Biochem. 2018; 55:165–71. 10.1177/0004563217699233 28504609

[25] Aregbesola A, de Mello VD, Lindström J, Voutilainen S, Virtanen JK, Keinänen-Kiukaanniemi S, Tuomainen TP, Tuomilehto J, Uusitupa M. Serum adiponectin/Ferritin ratio in relation to the risk of type 2 diabetes and insulin sensitivity. Diabetes Res Clin Pract. 2018; 141:264–74. 10.1016/j.diabres.2018.05.012 29777745

[26] Bu Y, Okunishi K, Yogosawa S, Mizuno K, Irudayam MJ, Brown CW, Izumi T. Insulin Regulates Lipolysis and Fat Mass by Upregulating Growth/Differentiation Factor 3 in Adipose Tissue Macrophages. Diabetes. 2018; 67:1761–72. 10.2337/db17-1201 29945891

[27] Weber M, Sporrer D, Weigert J, Wanninger J, Neumeier M, Wurm S, Stögbauer F, Kopp A, Bala M, Schäffler A, Buechler C. Adiponectin downregulates galectin-3 whose cellular form is elevated whereas its soluble form is reduced in type 2 diabetic monocytes. FEBS Lett. 2009; 583:3718–24. 10.1016/j.febslet.2009.10.008 19818774

[28] Miao Z, Lin JS, Mao Y, Chen GD, Zeng FF, Dong HL, Jiang Z, Wang J, Xiao C, Shuai M, Gou W, Fu Y, Imamura F, et al. Erythrocyte n-6 Polyunsaturated Fatty Acids, Gut Microbiota, and Incident Type 2 Diabetes: A Prospective Cohort Study. Diabetes Care. 2020; 43:2435–43. 10.2337/dc20-0631 32723842 PMC7510039

[29] Lindström J, Tuomilehto J. The diabetes risk score: a practical tool to predict type 2 diabetes risk. Diabetes Care. 2003; 26:725–31. 10.2337/diacare.26.3.725 12610029

[30] Wilson PW, Meigs JB, Sullivan L, Fox CS, Nathan DM, D’Agostino RB Sr. Prediction of incident diabetes mellitus in middle-aged adults: the Framingham Offspring Study. Arch Intern Med. 2007; 167:1068–74. 10.1001/archinte.167.10.1068 17533210

[31] American Diabetes Association. 2. Classification and Diagnosis of Diabetes: Standards of Medical Care in Diabetes-2019. Diabetes Care. 2019 (Suppl 1); 42:S13–28. 10.2337/dc19-S002 30559228

